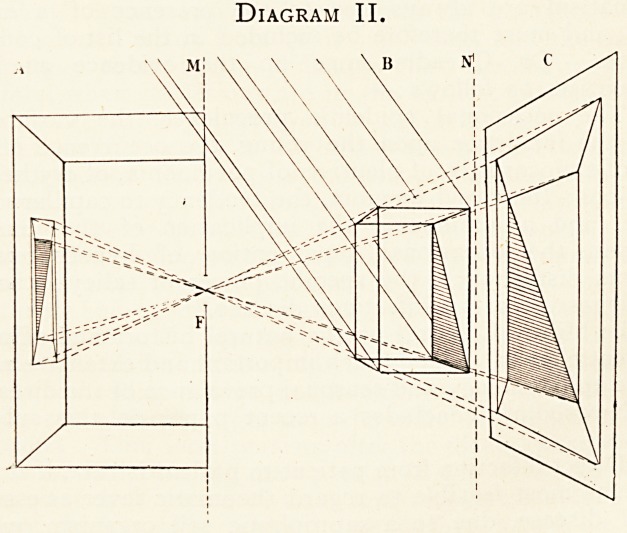# X-Ray Photographs as Pictures

**Published:** 1901-06

**Authors:** William Cotton


### X-RAY PHOTOGRAPHS AS PICTURES:

### WITH TWO DIAGRAMS.

BY

William Cotton, M.A., M.D., D.P.H.

---

Before X-ray photographs can be correctly interpreted it is necessary to settle how far as pictures (or "plane delineations") they agree with, or differ from, ordinary photographs.

In this comparison it is well to keep in mind at the outset that refraction by a lens is not indispensable to the formation of a photographic image. The effective range of fluorescent and actinic X-rays is limited to two or three feet, and for this distance from a camera a "pinhole" in a metal disc does quite as well in ordinary photography as any lens or combination of lenses, so far as pictorial effect and clearness are concerned, provided the exposure is sufficiently prolonged. The focus of a "pinhole" camera is the crossing station for reflected rays; the centre of the anti-cathodal plate of the vacuum tube is a focus of origin, and fairly comparable in size with the other. Nor is there any particular mystery about the use of the camera; some such device would have to be used in X-ray work if more than one source of X-rays were simultaneously in action near the plate.

With ordinary photography, by the crossing at the

"pinhole" of the camera of single rays reflected from each point of an external illuminated body there is formed on the ground glass screen or dry-plate a visible or a latent image respectively. This image is in proper gradation of tone according to the value impressed on each ray at the point of reflection. Viewed from the front of the screen (or from the film side of the developed "negative" as it lay in the camera, according to ordinary practice), the image is found to be inverted, reversed as regards right and left, in perfect perspective, and, as a rule, of smaller dimensions than the object depicted. When the "negative" is turned upside down and looked at through its smooth glass side at a distance from the eye equal to its original distance from the "pinhole"—or the corresponding "print" may be viewed from its face—every part is seen under the same angle as it appears under in the objects pourtrayed, point for point, the same as in an accurate drawing.

With X-rays, by their divergence from the anti-cathodal centre of emission, a visible or a developable image, as the case may be, is thrown on the fluorescent screen or photographic plate of any intervening bodies, in a gradation of tone depending inversely on the hindrance of each ray by the structures in its path. Viewed from the side of the screen or plate nearer to the centre of emission, the image is found to be erect, and with the same order of parts (left to right and right to left) as in the object itself. The same essential rules of perspective hold as in the other case. The image is larger in its parts necessarily than the object, though of course opaque lines X-rayed in contact with the plate are of natural dimension. When the "negative" is held in front of the eye at a distance equal to the original distance from the focus point, and with the film side towards the observer, every part appears under the same angle as it would appear under in the actual object, supposing it were correspondingly visually transparent without refraction.

Many of these points and the modes of reasoning about them are illustrated in the perspective Diagrams I. and II.:—

I. A, B, and C are parallel planes. B is transparent, and has drawn on it in opaque lines an easily recognisable figure a b c; a b c is copied as a "reflection image" $a^e b^e c^e$ on A in a camera with a "pinhole" at F; a b c can also be copied as a "projection image" (or shadow) $a^x b^x c^x$ on C when F is either an X-ray centre of emission or a small luminous flame. A may be an ordinary screen viewed from the side adjacent to F, or a "negative" looked at from its film side and from the same position. C may be a fluorescent screen viewed from the side nearest F or a developed dry-plate similarly regarded, or it may be an ordinary white screen. Again, if F be the position of a draughtsman's eye looking towards B, then he could draw a picture of a b c on some parallel plane between his eye and B. But before $a^e b^e c^e$ can be got to correspond with a b c we must invert A and look through it from the back (or look at a "print" from it). No such corrections are needed with C, though $a^x b^x c^x$ from the divergent nature of the projection is necessarily larger than a b c.

II. All to the left of the dotted line N indicates the formation of the "reflection image"; all to the right of the dotted line M indicates the formation of a "projection image." A, C, and F are as in I. B is a cube of wire to serve as an object to be taken on A or C. The side of B remote from F is distinctively marked to correspond with a b c in I., and to facilitate comparison of the perspective delineations on A and C. The images on A and C are geometrically similar to each other; but that on A cannot be pictorially compared with B or that on C (owing to the inversion and reversal of that on A) until A has been re-inverted and looked at through the back, or until in the ordinary way a "print" has been taken from it. The "print" from C would correspond with the image on A, barring inversion and size, but be equally fallacious in regard to order of parts.

In actual experimentation with X-rays solid wooden blocks dusted with subnitrate of bismuth are convenient to use instead of skeleton wire figures. The near and far sides from F are distinguished by letters written in an ink impervious to X-rays—*e.g.*, subnitrate of bismuth suspended in gum. Best of all is the hand, with the skin of the palm and fingers similarly dusted, to bring out the surface contours. A right hand X-rayed from F with palm towards C comes out perspectively on C as a right hand viewed from the back; in a "print" from C it is a left hand viewed from the back—both enlarged. A right hand taken with its dorsum towards C comes out perspectively on C (*i.e*, on the "negative" viewed from its face) as a right hand viewed through the palm, and in the "print" as a left hand viewed through the palm. At least these are what should be looked for in the respective instances, and not any other combinations of side and aspect. And so on, no doubt, with other parts of the body, paradoxical as it may seem to those who are misled by the false analogy between ordinary and X-ray prints.

For some time I have been investigating similar problems (more especially in relation to the stereoscope and the illusion known as "conversion of relief" in X-ray work) with the practical collaboration of Mr. Thomas Clark, and hope some day to lay the results more fully before those interested. I wish in the meantime to place on record the following conclusions:—

(1) An X-ray photograph is pictorially as much a photograph as any other of near objects.

(2) The perspective is the same as in any ordinary drawing, the only difference being that the plane of delineation is on the far side of the object depicted from the eye.

(3) An X-ray "negative" viewed from its face corresponds in order of parts with the object depicted; the "print" therefore, unless viewed in a mirror or rendered transparent and looked at through its back, should be discarded.

(4) As yet the eye (unaided by the stereoscope) does not properly appreciate relief in the single X-ray photograph; nevertheless, that aspect of the object pourtrayed that was actually nearest the X-ray focus at the time of taking, is to be looked for as nearest the eye.

(5) For medico-legal or other precise purposes no single X-ray photograph is of full value unless it has marked on it—

(*a*) Whether it is a "negative" or "print."

(*b*) The part represented.
(*c*) The side of the body—right or left.
(*d*) The part of the region represented nearest the plate.
(*e*) The position of the focus, or anti-cathodal centre of emission, in relation to the plate.

DIAGRAM I.

DIAGRAM II.

## Figures and Tables

**Diagram I. f1:**
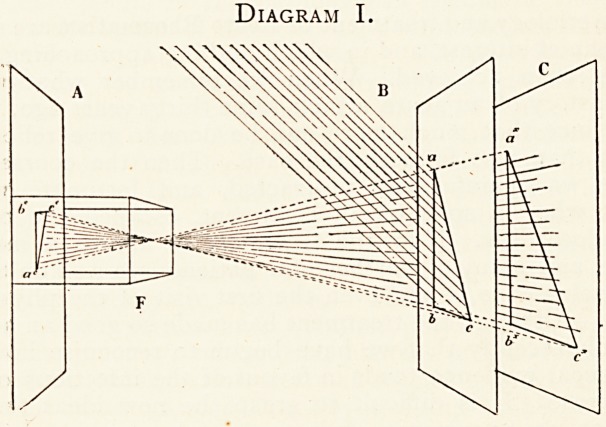


**Diagram II. f2:**